# Physical and Performance Profiles Differentiate Competitive Levels in U-18 Basketball Players

**DOI:** 10.3390/sports14010027

**Published:** 2026-01-05

**Authors:** Anna Goniotaki, Dimitrios I. Bourdas, Antonios K. Travlos, Panteleimon Bakirtzoglou, Apostolos Theos, Emmanouil Zacharakis

**Affiliations:** 1 School of Physical Education and Sports Science, National and Kapodistrian University of Athens, 41 Ethnikis Antistasis, 17237 Athens, Greece; goniotakia@yahoo.gr (A.G.); emzach@phed.uoa.gr (E.Z.); 2 Section of Sport Medicine & Biology of Exercise, School of Physical Education and Sports Science, National and Kapodistrian University of Athens, 41 Ethnikis Antistasis, 17237 Athens, Greece; dbourdas@phed.uoa.gr; 3 Department of Sports Organization and Management, Faculty of Human Movement and Quality of Life Sciences, University of Peloponnese, Efstathiou and Stamatikis Valioti & Plataion Avenue, 23100 Sparta, Greece; atravlos@uop.gr; 4 School of Physical Education and Sport Science, Aristotle University of Thessaloniki, University Campus, 54124 Thessaloniki, Greece; bakirtzoglou@phed.auth.gr; 5 Section of Sports Medicine, Department of Community Medicine and Rehabilitation, Umeå University, Biologihuset hus C, 901 87 Umeå, Sweden; 6 Umeå School of Sport Sciences, Umeå University, 901 87 Umeå, Sweden

**Keywords:** profiling and monitoring, skill development, talent identification, anthropometric, physiological–technical parameters, adolescent–young–youth, physical fitness, aerobic–anaerobic

## Abstract

Background: Evidence on how physical and technical factors distinguish U-18 basketball levels is limited, yet these determinants may aid talent identification and development. This study examined differences in anthropometric, physical performance, and technical characteristics between high-level (HL; n = 38) and low-level (LL; n = 35) U-18 male basketball players and explored relationships between technical skills and key physical attributes across all participants. Methods: Participants were evaluated across anthropometry, physical performance, and basketball-specific technical skills. Statistical analyses assessed between-group differences and correlations, with significance set at *p* ≤ 0.05. Results: Compared to LL players, HL players exhibited significantly superior physical attributes, including greater height (Cohen’s d = 0.67) and arm-span (d = 0.65), reduced body fat (d = −0.58), and advanced performance metrics (10 m-speed running (d = −0.78), 20 m-speed running (d = −0.93), flexibility (d = 1.26), counter-movement jump height (d = 1.27), intermittent endurance (d = 1.18)). Technical proficiency in tasks such as 10 m- and 20 m-speed dribbling, maneuver dribbling and defensive sliding was also significantly faster in the HL group (d = −0.96, d = −1.05, d = −1.87, and d = −1.14, respectively). Several anthropometric and performance variables were strongly correlated with technical skills, indicating their relevance for distinguishing competitive levels. Conclusions: These findings underscore the interplay of physical, technical, and performance factors in high-level youth basketball. Coaches may use this information to guide targeted training strategies that support talent identification, player development, and competitive success.

## 1. Introduction

Basketball is a dynamic [1,2] and widely popular team sport [3] that demands a multifaceted skill set encompassing technical, tactical, psychological, and physiological attributes [4,5,6,7,8]. Physiologically, basketball is characterised by an intermittent pattern of brief, high-intensity actions (e.g., sprints, jumps, and rapid defensive movements lasting 2–5 s) interspersed with longer periods (5–15 s) of low-intensity activity, including walking and jogging [1]. Time–motion analysis in elite male players has indicated that they perform on average about 105 high-intensity runs per game (mean duration ≈ 1.7 s, i.e., roughly one every 21 s of live time), with ≈60% of live time spent in low-intensity activities (standing, walking, jogging) and ≈15% in high-intensity activities [9]. Similarly, in elite under-19 (U-19) players, approximately 8.8% of live time is devoted to high-intensity specific movements, 5.3% to sprinting, and 2.1% to jumping, highlighting the frequent but very short explosive efforts embedded within predominantly lower-intensity movement patterns [10]. These physical demands are complemented by anthropometric and technical skill profiles, which play an essential role in differentiating player performance and competitive levels [2,4,8,11].

Among U-18 basketball players, significant variability exists in anthropometric characteristics based on playing positions and competitive levels [10,12,13]. Notably, elite youth players tend to be taller and reach their peak height velocity earlier than their sub-elite counterparts [14], underscoring the influence of maturation on performance in youth basketball [15]. Beyond differences in stature and mass, biological maturity (e.g., skeletal age relative to chronological age) and relative age within the selection year are particularly important in cadet and junior categories. Around puberty, the timing of peak height velocity can differ by approximately four to five years between early- and late-maturing boys, and early-maturing players systematically display advantages in anthropometric characteristics and strength–power capacities [16]. Consistent with this, studies in elite adolescent male basketball players report both a predominance of early-maturing athletes and an over-representation of those born in the first half of the competitive year among selected squads [14,17]. Recent large-scale data from elite U14–U17 male basketball players in China further showed that approximately 59–61% of selected athletes were born in the first six months of the year, with 35–39% born in the first quarter alone, indicating a clear relative age effect that favours players born earlier in the selection year and likely contributes to the anthropometric and performance variability observed in U-18 cohorts [18]. Despite prior work describing general anthropometric characteristics in youth basketball, limited evidence specifically compares these attributes between U-18 athletes competing at distinct competitive levels [19]. Existing studies predominantly focus on younger age groups or mixed cohorts, leaving a gap in understanding how maturational status, fitness-related and key anthropometric factors differentiate late-adolescent players who are already undergoing or nearing biological maturation [13]. Such comparisons are important for identifying attributes associated with higher competitive placement, informing talent identification, and guiding age-appropriate training and development strategies. Focusing specifically on the U-18 category is particularly relevant because this stage represents a transitional period in which athletes have typically completed or nearly completed biological maturation, thereby reducing maturity-related biases that complicate comparisons in younger age groups (e.g., U-14 or U-16) [8]. The U-18 level also corresponds to a critical developmental phase in which players prepare for entry into professional academies, collegiate programmes, or national pathways, making the identification of performance determinants especially valuable for talent selection and long-term athlete development. Additionally, U-18 athletes experience greater specialisation and higher training loads than younger cohorts, which may accentuate differences in anthropometric, physical performance, and technical characteristics across competitive levels [20,21]. Despite this, very few studies have examined competitive-level distinctions specifically within this age category, limiting our understanding of the attributes associated with success at this crucial stage.

Early engagement in basketball, the time invested in practice, and competitive experience positively influence player performance [22]. However, it is unclear whether these factors impact U-18 players differently depending on their competitive category. Similarly, physical performance traits, such as explosive power, are crucial to basketball success [23,24]. Adult and U-19 basketball players have been shown to perform an average of 46 and 44 jumps per game, respectively [9,10,25], indicative of the demand for lower-limb explosive power. Time–motion analysis in elite U-19 players further indicates that approximately 16% of live playing time is spent in high-intensity activities, with about 8–9% devoted to high-intensity basketball-specific movements such as defensive shuffling, cuts and accelerations, around 5% to sprinting, and about 2% to jumping [10]. In addition, across men’s and women’s elite competitions players typically cover ≈5–6 km during a 40 min game and frequently alternate between low-intensity movements (standing, walking, jogging) and short bursts of high-speed running and shuffling, changing movement pattern (e.g., running, lateral shuffling, jumping) every ~1–3 s [1,26]. Consistent with these demands, higher-level adult players demonstrate superior speed, agility, vertical jump capacity, upper-limb strength, and knee extension torque compared to their lower-level peers [27,28,29]. Furthermore, aerobic fitness in elite junior basketball players surpasses that of sub-elite players [30]. Nevertheless, it remains uncertain whether specific fitness and physical performance characteristics—such as handgrip strength, flexibility, leg power, sprinting, and aerobic capacity—differ significantly between U-18 players competing at distinct levels of play.

Proficiency in technical basketball skills—such as shooting, passing, dribbling, and defensive sliding—is fundamental to performance and competitive success in modern basketball [2,8,31,32,33]. These skills directly reflect a player’s ability to execute game-relevant actions under time pressure, defensive constraints, and dynamic tactical conditions. Dribbling speed, maneuver dribbling, and defensive sliding are particularly sensitive to differences in neuromuscular coordination, decision-making under movement demands, and exposure to advanced training environments, while shooting and passing accuracy represent core indicators of technical execution and game readiness [7,11,21]. Although previous studies have shown that ball-handling speed and shooting accuracy can distinguish elite from sub-elite youth players [11,14], limited research has specifically examined whether these technical components differentiate U-18 athletes competing at different competitive levels. The selected assessments therefore target essential, position-independent skills that are expected to reveal performance disparities relevant to this age group and competitive context [8,20].

Basketball leagues are typically stratified into hierarchical divisions that delineate competitive levels. Research investigating how anthropometric, physiological, performance, and technical skill parameters interact to distinguish U-18 basketball players competing at different tiers is scarce. Studying these variables collectively can provide valuable insights into the determinants of success in basketball [2,8], potentially enhancing talent identification, player development, and coaching strategies. This study aimed to investigate differences in anthropometric, physical performance, and technical parameters between U-18 basketball players competing at higher and lower competitive levels. We hypothesised that players at higher levels would outperform their lower-level counterparts across all measured variables. Furthermore, we anticipated significant relationships between basketball technical skills and anthropometric and physical performance variables across all participants.

## 2. Materials and Methods

### 2.1. Participants

Adolescent basketball players registered with 56 clubs (A and C leagues) in the Attica region of Greece were invited to participate via email communications sent to their respective clubs. A total of 121 athletes (112 males, 9 females) responded to the invitation. Despite efforts to recruit a sufficient number of female players with comparable competitive backgrounds, non-responsiveness and declined participation resulted in insufficient female representation; thus, only male participants were included in the present study.

**Figure 1:**
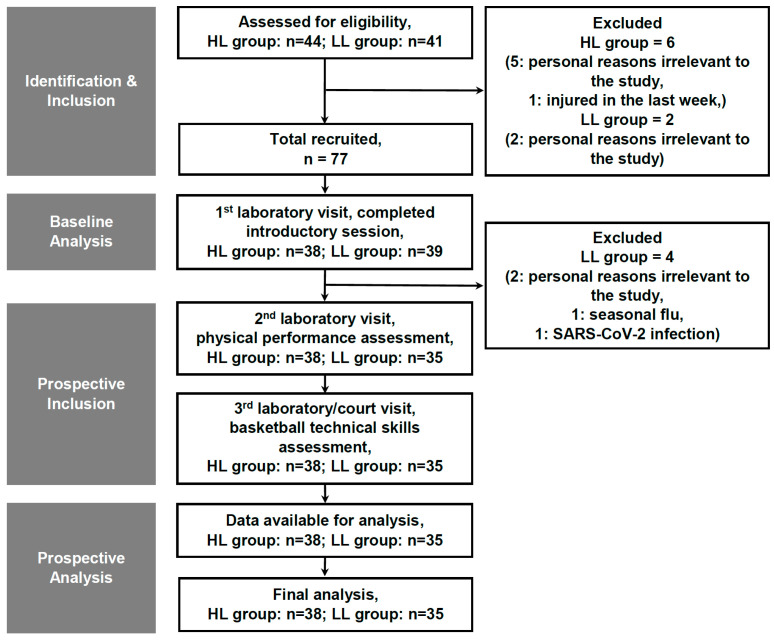
Flowchart depicting participant recruitment, group assignment, and advancement through the study phases. Abbreviations—HL: higher-level; LL: lower-level; n: sample size.

From the 112 male respondents, 85 players were randomly sub-selected using Excel’s RANDBETWEEN function to ensure equal probability of inclusion among those who volunteered to participate. No a priori power analysis was conducted due to the unpredictable number of volunteer respondents; however, a post hoc power analysis was performed and is reported in Section 2.5 (Statistical Analyses). Following eligibility screening—based on predefined inclusion criteria (male adolescent basketball players aged 15–17 years; habitually active, defined as engaging in ≥60 min of moderate-to-vigorous physical activity daily; exclusive participation in basketball; no musculoskeletal injury within the previous six months; ≥1 year of basketball training experience; adequate sleep habits (≥8 h per night); completion of parental/guardian screening questionnaires assessing physical activity and sleep patterns; and medical clearance)—and exclusion criteria (basketball experience <1 year; visual, vestibular, or neuromuscular impairments; inadequate sleep <8 h per night; significant medical conditions; active medication use; history of smoking or alcohol consumption; or incomplete data), eight players were excluded. The final sample therefore consisted of 73 adolescent male basketball athletes (Figure 1).

Of these, 38 players competed in the higher-level (HL) U18 A league, while 35 competed in the lower-level (LL) U18 C league. All participants and their legal guardians provided written informed consent after receiving a detailed explanation of the study objectives, procedures, and potential risks. Participants were informed of their right to withdraw at any point without consequence. Parents or guardians completed validated screening questionnaires on physical activity and sleep habits [34,35,36], and all players underwent a comprehensive medical evaluation prior to participation. This study employed a cross-sectional observational design and received ethical approval (1173/11-03-2020) from the university’s research ethics committee. All ethical procedures adhered to the Declaration of Helsinki [37].

### 2.2. Procedures

Participants and their legal guardians were not involved in the study design or data processes; athletes only made minor training schedule adjustments to accommodate the testing protocol. The research team was gender-diverse and comprised early-career and senior researchers from institutions in Greece and Sweden, with interdisciplinary expertise in sports medicine, exercise physiology, sport science, statistics, and strength and conditioning.

At the start of the study, participants attended an orientation session in which they were familiarised with the laboratory and court environments and received a detailed explanation of all procedures. Anthropometric measurements—including height (Stadiometer®, Seca, Birmingham, UK), sitting height, leg length, arm-span (Height Indicator Tape Ruler, Posh Rulers, London, UK), body mass (Beam Balance 710, Seca, Birmingham, UK), and skinfolds (Harpenden Skinfold Calipers, Baty International, Burgess Hill, West Sussex, UK)—were obtained twice, with a third measurement taken if discrepancies exceeded 4 mm (height/sitting height), 2 mm (skinfolds), or 0.4 g (body mass); the median or mean was used accordingly [38]. Body fat percentage was estimated from triceps and calf skinfolds using a sex- and pubertal stage-specific equation [39]. Maturity offset was calculated using established anthropometric prediction equations [38]. Hand dominance was determined based on self-reported preferred shooting hand. Participants were then introduced to the physical performance and basketball skill assessments.

One week later, participants returned for five physical performance tests, followed by a third visit one week afterward for five basketball skill tests. All assessments were performed on an official indoor hardwood court (28 × 15 m) under controlled environmental conditions (23–25 °C, 1010–1040 mmHg barometric pressure, ~51% humidity). Measurement devices were calibrated according to manufacturer guidelines before each session.

To minimise confounding factors, participants refrained from strenuous activity before testing [40,41] and avoided supplements with ergogenic effects [42,43,44]. A standardised evening meal and an overnight fast preceded each testing day. Testing began at 8:00–8:30 a.m. After arrival and bladder emptying, participants completed a standardised 20 min general warm-up consisting of light jogging, dynamic stretching, and high-intensity drills.

Participants were blinded to performance results and instructed not to share study procedures to reduce expectancy bias. Assistant researchers and participants were also unaware of the study’s specific aim. Testing took place in October, early in the competitive season, when accumulated match fatigue was expected to be minimal [45,46]. All participants were healthy, abstained from caffeine and alcohol, and avoided stimulant-containing medication throughout the study.

### 2.3. Physical Performance Evaluation

The physical performance tests were conducted sequentially in a fixed order (hand-grip strength → flexibility → countermovement jump → sprint → intermittent endurance), with a 10 min recovery period between assessments. All tests selected for this study have demonstrated good-to-excellent test–retest reliability in physically active and team-sport populations. Maximal hand-grip strength assessed with a hand-held dynamometer shows excellent reliability (ICC ≈ 0.96 over 7 days; [47]). Sit-and-reach flexibility demonstrates good reliability (ICC = 0.92; CV = 8.7%; [48]). Countermovement jump performance obtained using portable systems or force platforms yields ICC values between ~0.70 and 0.97 and CV values between ~1.8% and 11.0% in team-sport athletes [49,50]. Similarly, 5–20 m sprint times recorded with electronic timing gates demonstrate ICC = 0.71–0.90 and CV = 1.5–2.8% in elite court-sport athletes [50]. A systematic review of Yo-Yo intermittent endurance tests, including level 1, reports ICC values typically between 0.80 and 0.95 and CV < 10–11% in youth and adult team-sport players [51].

#### 2.3.1. Hand-Grip Strength

Hand-grip strength was measured using a Takei T.K.K.5401 GRIP-D dynamometer (Takei Scientific Instruments Co., Ltd., Tokyo, Japan) as per the procedure detailed by Gatt et al. [52]. Participants completed one familiarization trial and two formal attempts with each hand. The best result for each hand was recorded for analysis.

#### 2.3.2. Flexibility of the Lower Back and Hamstrings

After a 10 min standardized warm-up, consisting of light jogging and dynamic and static whole-body stretches, flexibility of the lower back and hamstrings was assessed using a sit-and-reach box (60 × 35 × 35 cm; Cranlea Human Performance Limited, Birmingham, UK). Participants removed their footwear and sat with legs fully extended, placing their soles flat against the box. With knees locked and pressed against the floor, they reached forward with stacked palms facing downward along the measurement scale. The furthest reach distance, measured with both hands moving simultaneously, among three trials with a one-minute rest interval in between, was recorded [53].

#### 2.3.3. Vertical Jump

Following a brief 5 min warm-up on a leg cycle ergometer at 60–70% intensity (894E®, Monark, Varberg, Sweden) [54], participants performed the counter-movement jump with arm swing to evaluate lower limb power. Using an infrared timing system (ERGO JUMP Plus–BOSCO SYSTEM, Byomedic, S.C.P., Barcelona, Spain), participants executed three vertical jumps from a 90° squat position with their torso upright and hands on the waist. Each jump was separated by one minute of rest. The highest recorded value was used for analysis [55].

#### 2.3.4. Sprint Performance

The sprint test was performed on a 20 m straight-line track with split times recorded at 10 m. Times were measured with infrared light sensors placed at a height of 1 m (Racetime 2, Microgate, Bolzano, Italy). Participants began running from a standing position, ensuring the front foot was 30 cm behind the start line. After a practice trial, they performed two timed attempts separated by a one-minute rest. The best result was recorded.

#### 2.3.5. Intermittent Endurance

Intermittent endurance was assessed using the Yo-Yo Intermittent Endurance Level 1 (Yo-Yo IE1) test [56], which is associated with aerobic response and endurance capacity [57]. This test involved two 20 m shuttle runs at progressively increasing speeds, interspersed with 5 s active recovery periods during which participants walked or jogged 2.5 m past the finish line marker. Participants started at an initial running speed of 8 km·h^−1^, with pacing controlled by auditory signals from a CD player (AZB798T/12, Philips, Amsterdam, Holland). The test continued until participants were unable to meet the required pace on two consecutive occasions or voluntarily withdrew due to fatigue. The total distance covered was recorded, and heart rate data were telemetrically monitored every 5 s using a Polar heart rate monitor (Polar Sport Tester, Polar Electro Oy, Kempele, Finland).

### 2.4. Assessment of Basketball-Specific Technical Skills

The basketball-specific skill tests were performed sequentially in a fixed order (shooting → passing → straight-line dribbling → maneuver dribbling → defensive sliding) with a 10 min recovery between assessments. All tests used an official FIBA size-7 ball (Precision TF-1000, Spalding, Keysborough, Australia). The basketball-specific technical skill tests used in this study have previously demonstrated strong measurement properties. According to Hopkins et al. [58], these tests show validity coefficients ranging from 0.73 to 0.91 and test–retest reliability coefficients (intraclass correlation coefficients; ICC) between 0.88 and 0.98 in youth basketball players.

#### 2.4.1. Shooting Speed and Accuracy Test

**Figure 2:**
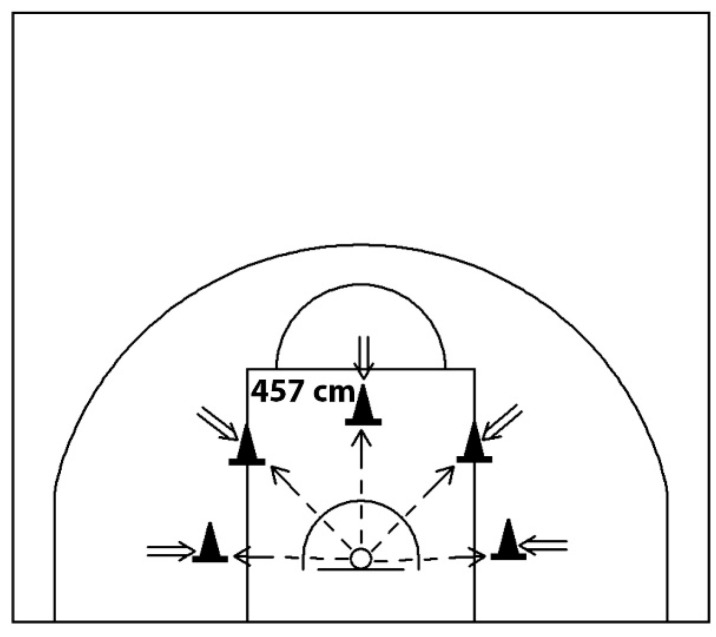
Speed and accurate shooting test from 4.57 m horizontal basket distance for 60 s.

This test (Figure 2) assessed players’ ability to shoot accurately at pace, following Hopkins et al. [58]. Participants shot from behind a 4.57 m line, using different locations for each attempt. Each trial lasted 60 s. After one familiarisation trial, two scored trials were completed with 3 min rest. Scoring awarded 2 points for made shots and 1 point for rim contact. The sum of the two scored trials was used for analysis.

#### 2.4.2. Passing Accuracy Test

**Figure 3:**
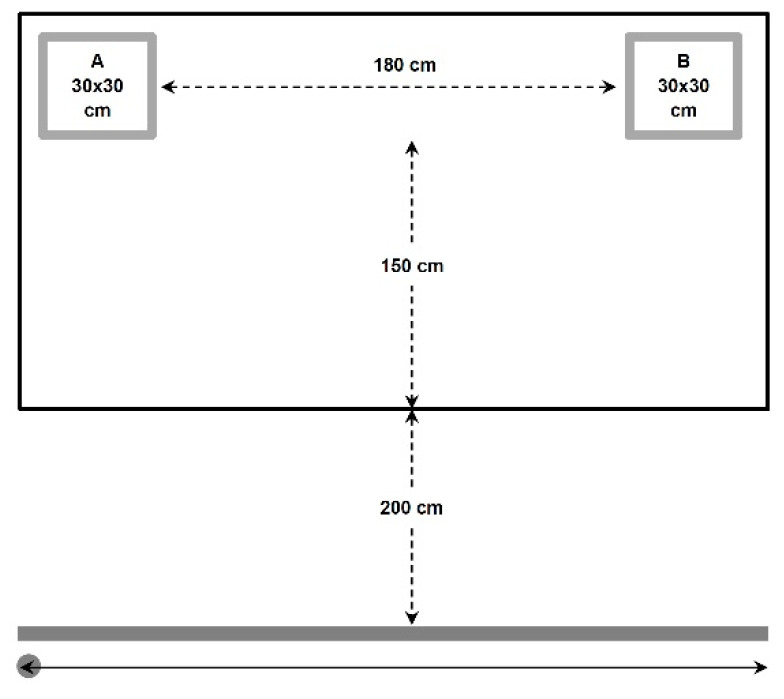
Passing accuracy test. The subject started the attempt from the left side, passing with two hands from the chest on target (A). Then received the ball and moved on to the second (B) target, where also passing and receiving was required, then to the target (A), and so on.

Based on Hopkins et al. [58], participants passed against two 30 × 30 cm targets on a wall while moving laterally (Figure 3). After one familiarisation trial, two 30 s scored trials were completed with 3 min rest. One point was awarded for passes striking the target or border. The best of the two trials was retained.

#### 2.4.3. Straight-Line Dribbling Test, (Right/Left Hand)

This test evaluated controlled dribbling speed over 20 m. Split times at 10 m were measured using infrared timing gates (Racetime 2, Microgate, Bolzano, Italy). Participants performed one familiarisation attempt per hand, followed by two scored trials (right/left). The mean of the two trials was used for analysis.

#### 2.4.4. Maneuver Dribbling Test, (Right/Left Hand)

**Figure 4:**
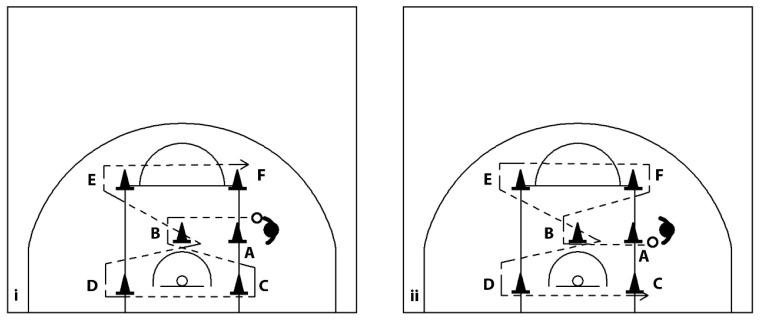
Maneuver dribbling test, (**i**) right and (**ii**) left hand. Participants had to complete a dribbling task over a set distance, navigating six (6) obstacles as quickly as possible, starting from point A and finishing at point F.

Following Hopkins et al. [58], participants dribbled through six cones while alternating hands (Figure 4). One familiarisation trial preceded two scored trials, starting with opposite hands and separated by 3 min rest. The average completion time was used for analysis.

#### 2.4.5. Defensive Sliding Test

**Figure 5:**
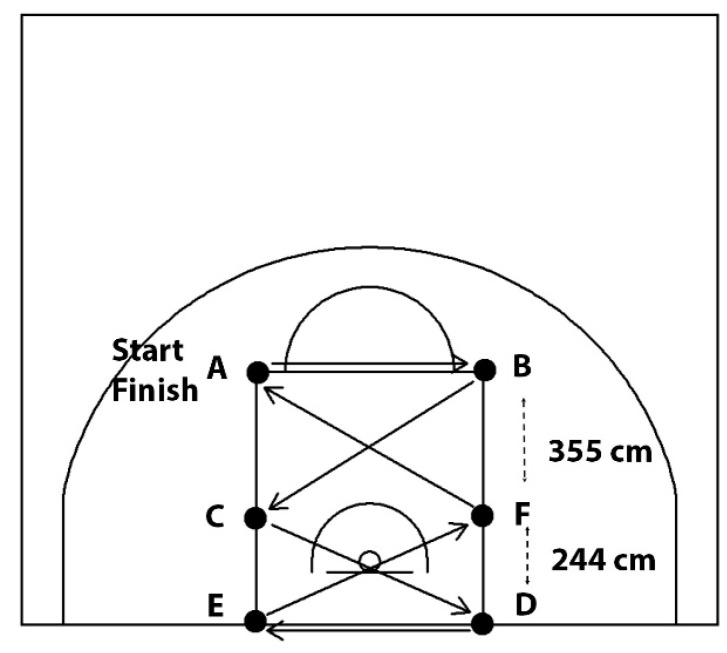
Defensive sliding test. Participants had to perform a lateral slide as quickly as possible, starting from point A, passing through five intermediate points (B–F), and finishing back at point A.

Adapted from Hopkins et al. [58], this test (Figure 5) assessed lateral defensive agility. Participants slid laterally between designated points, touching the ground at each turn without crossing legs. After one familiarisation attempt, two scored trials were performed with 3 min rest. The mean of the two valid trials represented the final score.

### 2.5. Statistical Analyses

The dataset was examined for normal distribution and equality of variances using the Shapiro–Wilk test and Levene’s test, respectively, ensuring that both assumptions were satisfied (*p* > 0.05). To compare mean values for anthropometric, physical performance, and basketball technical skill parameters between groups defined by competitive level (higher-level versus lower-level leagues), independent *t*-tests were conducted [59]. Pearson’s correlation coefficient (r) was utilized to evaluate the strength and direction of relationships between physical performance measures and technical skill variables across all participants. The interpretation of effect sizes (Cohen’s d (d)) followed established conventions, classifying correlations as small (r ≈ 0.10), medium (r ≈ 0.30), or large (r ≈ 0.50) according to Cohen [60]. All analyses were performed using IBM SPSS Statistics (version 29.0, IBM Corp., Armonk, NY, USA), with statistical significance set at α = 0.05 for all tests. Descriptive statistics, including means ± standard deviations (SD) and 95% confidence intervals (CI) where applicable, were reported unless stated otherwise.

Additionally, a post hoc power analysis was performed using the G*Power software (version 3.1.9.2, Heinrich-Heine-University, Düsseldorf, Germany). This analysis focused on the 10 m sprint as the primary criterion variable and utilized the following parameters: effect size (d) = 0.778, significance level (α) = 0.05, and a design involving two independent groups (HL: n_1_ = 38; LL: n_2_ = 35) in a single condition. The resulting observed power (1–β) was determined to be greater than 0.94. Comparable power estimates were obtained when analyzing other variables such as hamstring and lower back flexibility, counter-movement jump, 20 m sprint, Yo-Yo IE1, 10 m dribbling, 20 m dribbling, maneuver dribbling, and defensive sliding.

## 3. Results

**Figure 6:**
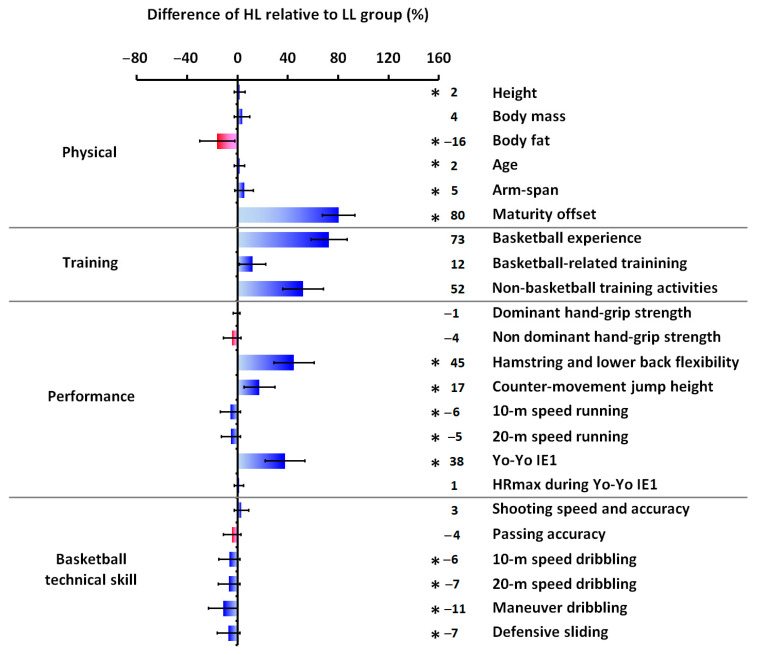
Percentage difference calculated as [(HL−LL)/LL] × 100 for physical, basketball training, performance, and basketball technical skill parameters. The * indicates a statistically significant difference between HL and LL groups at *p* ≤ 0.05, and regardless of the statistical significance and the positive or negative sign, blue color bars indicate only better performance/basketball technical skill or greater value in the HL group, whereas red color bars indicate better performance/basketball technical skill or greater value in the LL group. Error bars present the lower and upper bounds of the 95% CI. Abbreviations—CI: confidence interval; HL: higher-level; HRmax: maximal heart rate; LL: lower-level; Yo-Yo IE1: Yo-Yo Intermittent Endurance Level 1 test.

**Figure 7:**
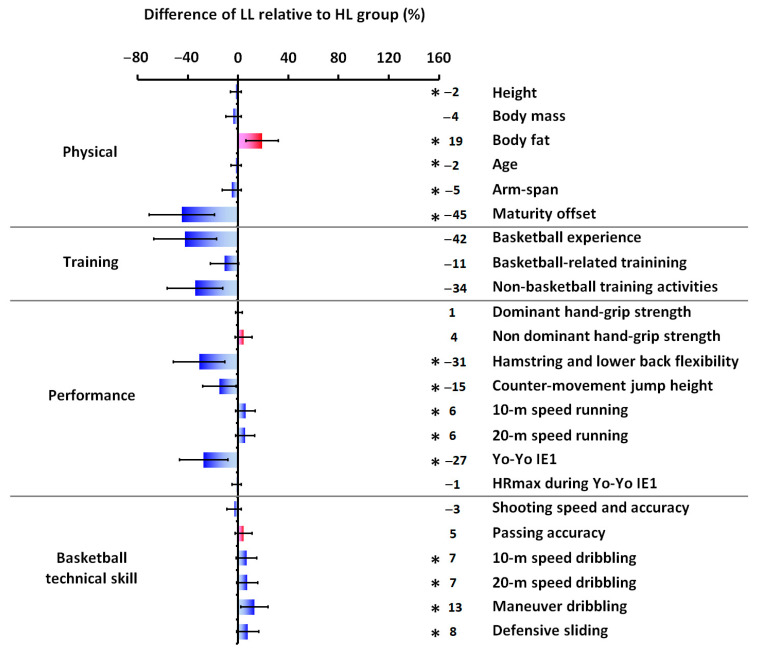
Percentage difference calculated as [(LL−HL)/HL] × 100 for physical, basketball training, performance, and basketball technical skill parameters. The * indicates a statistically significant difference between HL and LL groups at *p* ≤ 0.05, and regardless of the statistical significance and the positive or negative sign, blue color bars indicate only better performance/basketball technical skill or greater value in the HL group, whereas red color bars indicate better performance/basketball technical skill or greater value in the LL group. Error bars present the lower and upper bounds of the 95% CI. Abbreviations—CI: confidence interval; HL: higher-level; HRmax: maximal heart rate; LL: lower-level; Yo-Yo IE1: Yo-Yo Intermittent Endurance Level 1 test.

Anthropometric and basketball activity profile data for each group and overall are provided in Table 1, while Table 2 summarizes the performance and basketball technical skill data, respectively. The HL group demonstrated superior performance compared to the LL group across several variables. Significant differences (*p* ≤ 0.001) favoring the HL group were observed in hamstring and lower back flexibility (t(71) = 5.480, d = 1.26), counter-movement jump height (t(71) = 5.431, d = 1.27), 10 m sprint time (t(71) = −3.364, d = −0.78), 20 m sprint time (t(71) = −3.879, d = −0.93), and Yo-Yo Intermittent Endurance Level 1 test performance (t(71) = 5.040, d = 1.18). The HL group also exhibited superior performance in basketball technical skills, including 10 m dribbling time (t(71) = −4.275, d = −0.96, *p* ≤ 0.001), 20 m dribbling time (t(71) = −4.253, d = −1.05, *p* ≤ 0.001), maneuver dribbling performance (t(71) = −7.923, d = −1.87, *p* ≤ 0.001), and defensive sliding performance (t(71) = −4.861, d = −1.14, *p* ≤ 0.001). While no statistically significant differences were found between groups for basketball-related training (t(71) = 1.873, d = 0.44, *p* = 0.065) or non-basketball training activities (t(71) = 1.742, d = 0.42, *p* = 0.087), the HL group accumulated significantly more overall training hours (t(71) = 2.260, d = 0.54, *p* = 0.028). Furthermore, Figure 6 and Figure 7 depict the percentage differences in mean values between the groups for anthropometric characteristics, basketball experience, weekly training load, performance metrics, and basketball technical skills.

Pearson’s correlation coefficients between anthropometric, physical performance, and technical skill variables across all participants (N = 73) indicated large and significant correlations between basketball experience and both 10 m dribbling (r = −0.610, *p* ≤ 0.001) and defensive sliding (r = −0.564, *p* ≤ 0.001). Similarly, counter-movement jump height performance showed significant correlations with 20 m dribbling (r = −0.567, *p* ≤ 0.001) and defensive sliding (r = −0.520, *p* ≤ 0.001). Furthermore, 10 m sprint performance was significantly correlated with 10 m dribbling (r = 0.684, *p* ≤ 0.001), 20 m dribbling (r = 0.642, *p* ≤ 0.001), and defensive sliding (r = 0.524, *p* ≤ 0.001). Finally, 20 m sprint performance exhibited large correlations with 10 m dribbling (r = 0.622, *p* ≤ 0.001), 20 m dribbling (r = 0.856, *p* ≤ 0.001), and defensive sliding (r = 0.638, *p* ≤ 0.001).

## 4. Discussion

The primary purpose of this study was to identify notable differences in anthropometric, physical performance, and technical parameters between U-18 basketball players at high and low competitive levels. Main findings of this study are that higher-level players exhibited superior physical attributes, including greater height, arm-span, and lower body fat, alongside enhanced performance metrics such as speed, vertical jump, hamstring and lower back flexibility, and intermittent endurance. These results are consistent with our hypothesis that higher-level players would outperform their lower-level counterparts, likely due to a combination of training experience, maturation, and physical development. Advanced technical skills, particularly in dribbling and defensive sliding, further underscore the role of specialised training and experience in player development. These findings highlight the predictive value of physical and performance variables in distinguishing basketball skill levels, providing valuable insights into the multifaceted demands for competitive success.

Previous studies have demonstrated that adult basketball players competing at higher levels tend to be taller and possess longer limb lengths, traits often advantageous in basketball [17,61]. While this pattern was not evident in a study on Portuguese adolescent basketball players [62], it has been corroborated in various investigations involving adolescent and collegiate athletes across competitive tiers [14,17,61,63,64]. Conversely, differences in body mass across performance levels appear to be inconsistent in adolescent basketball players [14,62,63]. However, evidence suggests that higher-level team sport athletes typically exhibit lower body fat percentages [62,65]. In alignment with this literature, the findings from the present study reveal that players in the HL group display significantly greater height and arm-span compared to their lower-level counterparts, alongside a notably reduced body fat percentage. Furthermore, the observed maturity offset differences between the HL and LL groups are consistent with previous investigations involving youth basketball players at differing levels of competition [14,63]. Maturity offset, a key marker of biological age, profoundly influences growth, functional capacities, and athletic potential in young athletes [66]. Although early-maturing athletes may experience competitive advantages in youth basketball, it has been suggested that such differences may diminish following the completion of biological maturation [66,67]. Moreover, the HL group in this study demonstrated a training background (>5.5 yr) similar to that reported by Arede et al. [68], who found that extended training experience predicts higher competitive participation in young basketball players.

Basketball is an intermittent, high-intensity sport characterised by repeated short-duration sprints, rapid changes in direction, and frequent jumping, all of which rely primarily on phosphocreatine and anaerobic glycolytic pathways [1,2,69]. However, the cumulative duration of match play and the relatively high mean heart rate observed during competition (≈85% HRmax; [1]) indicate that aerobic metabolism contributes substantially to recovery between high-intensity actions and to sustaining repeated efforts [1,69,70,71]. In youth athletes, aerobic capacity continues to develop throughout adolescence, influenced by maturation, training exposure, and body composition [72,73]. Although HRmax did not differ between competitive levels in the present study, HL players covered a greater distance in the Yo-Yo IE1, suggesting superior intermittent endurance performance rather than directly measured aerobic capacity. This difference may reflect greater training volume and competitive exposure in the HL group, as well as their more favourable body composition profiles—specifically lower body fat percentage and higher lean mass—which are commonly associated with improved intermittent running performance [74,75]. These findings align with previous research reporting better Yo-Yo IE1 outcomes among higher-level youth basketball players [30].

Regarding upper limb strength, elite adult male basketball players have consistently shown superior strength compared to average-level players [29]. Similar trends have been observed in youth basketball, such as during the Portuguese U-14 National Championship, where finalists achieved significantly higher hand-grip strength than players from lower-ranked teams [62]. However, in this study, no significant differences in hand-grip strength were observed between the HL and LL groups, consistent with findings from other U-16 basketball study [63]. This lack of differentiation may reflect the prioritization of technical skill development (e.g., dribbling, shooting, passing) over targeted strength training at this developmental stage.

Muscle mass increases markedly between 13.5 and 15.5 years in males, with testosterone levels rising approximately tenfold during this period, significantly influencing muscle growth and performance [76]. Vertical jump performance, which improves linearly from childhood until early adolescence, continues to increase in males after 13–14 years, whereas it stabilizes in females [16,66]. In males, speed development exhibits a rapid acceleration during the ages of 7 to 10 years, attributed to neuromuscular maturation and enhanced motor coordination. This period of growth is followed by a deceleration phase and eventual stagnation around the age of 14 years, likely influenced by the interplay of hormonal changes and skeletal maturation [77]. In basketball, vertical jump and speed are critical performance traits, influencing activities such as rebounding, shooting, and fast breaks [78,79]. Although one study did not find significant differences in vertical jump performance between groups of different competitive levels [62], others have consistently shown higher counter-movement jump heights among higher-level youth basketball players [28,29]. The findings of this study align with previous research [62,63], as the HL group exhibited superior counter-movement jump heights and speed running compared to the LL group. Such differences may stem from variations in training volume, intensity, and specificity, as well as competitive pressures, early specialization, and selection processes [73,74,75,76].

Although flexibility is not considered a primary performance discriminator in basketball, it contributes to movement efficiency, power generation, and injury prevention [2,8,79]. In the present study, flexibility was significantly higher in the HL group, consistent with previous findings in adolescent basketball players [28,29,62], and this difference may partly reflect maturational influences, as early-maturing boys typically exhibit superior sit-and-reach performance and better motor fitness [80,81,82]. Given the magnitude of the between-group difference, potential measurement variability and individual differences should also be considered, so these results should be interpreted with caution.

In stop-and-go sports, higher-level athletes generally exhibit superior performance and a more extensive repertoire of movements and reactions compared to lower-level players [83,84]. In basketball, skills such as shooting, passing, dribbling, and maneuver dribbling serve as key differentiators between players of varying skill levels [2,8,14]. Higher-level players have been shown to excel in metrics such as successful 2-point shots, accurate passes, earned fouls, successful free throws, and the avoidance of turnovers [85]. The findings of this study corroborate previous evidence, with the HL group outperforming the LL group in defensive sliding, 10 and 20 m dribbling, and maneuver dribbling tests. These results align with prior research emphasizing the role of specialized training, increased competitive exposure, and early specialization in the development of these advanced motor and technical skills in higher-level athletes [86]. However, no significant differences were observed between the HL and LL groups in passing accuracy and shooting speed tests. This lack of significant differentiation in passing accuracy and shooting speed may be attributed to several factors, including individual variability, comparable training focus across groups, and potential limitations in the design or sensitivity of the testing protocols used [86]. These findings underscore the complex interplay of physical, technical, and training-related factors that influence performance differences across competitive levels in youth basketball.

In this study, the HL group outperformed the LL group in most anthropometric, training, performance, and technical variables. Consequently, it was anticipated that significant relationships would emerge among basketball technical skills and various anthropometric, training, and performance parameters. As expected, most anthropometric (e.g., height, body fat, age, arm-span), training (e.g., basketball experience, overall training volume), and performance (e.g., hand-grip strength, hamstring and lower back flexibility, countermovement jump height, 10 m and 20 m running, Yo-Yo IE1) variables were significantly correlated with at least three of the six assessed basketball technical skills. An exception was hand-grip strength, which showed a significant correlation only with passing accuracy. The strongest correlations identified included 10 m speed dribbling with basketball experience and 10 m and 20 m speed running; 20 m speed dribbling with countermovement jump height and short-distance sprinting; and defensive sliding with basketball experience, countermovement jump height, and sprint performance. These findings align with prior research on adolescent basketball players. For instance, Apostolidis et al. [87] showed significant correlations between height and arm-span with speed dribbling and maneuver dribbling. Additionally, hand-grip strength has been linked to speed spot shooting, speed dribbling, and maneuver dribbling [87]. Basketball experience has also been associated with technical skills such as passing, maneuver dribbling, and speed spot shooting [86], while basketball playing ability has been associated with speed running, flexibility, and hand-grip strength [88]. Moreover, previous study have identified a negative correlation between body fat percentage and various basketball technical skills, including maneuver dribbling, 28 m speed dribbling, shuttle running, and dribble shuttle running [89]. Players categorized as the “best” in their age group (U-16) tend to exhibit advanced maturity, larger stature, and superior fitness profiles compared to their peers [90]. However, the correlations observed in the present study should be interpreted with caution. Maturity status, training volume, and group-based differences likely contributed to shared variance among multiple variables, and the exploratory correlation analysis involved a large number of pairwise tests without adjustment for multiple comparisons. Given that the primary purpose of the study was to compare two competitive levels rather than to model causal determinants of performance, the relationships described above are descriptive in nature and should not be overinterpreted as independent predictors of technical skill. Nonetheless, the overall pattern reinforces the multifaceted nature of basketball performance, where physical attributes, experience, and performance capacities act jointly to influence technical proficiency and competitive success.

### Strengths, Limitations, Future Research Directions, and Practical Applications

This study provides important insights but is not without limitations. The participant sample was restricted to U-18 male basketball players from two competitive levels within a single region, and recruitment relied on a convenience sample followed by random sub-selection, which may introduce sampling bias and limit the generalisability of findings to broader populations. Additionally, technical skill assessments were performed under non-fatigued, controlled conditions, which do not fully reflect game-like demands. Although effect sizes were reported, no a priori power analysis was conducted, and thus the adequacy of the sample size cannot be fully guaranteed. Maturity offset differences between groups may also represent a confounding factor, and despite reporting maturity offset values, future studies should consider more precise biological maturation assessments. Further research should include more diverse samples across genders, age groups, and competitive levels; incorporate fatigue-based and sport-specific testing; and examine additional physical, psychological, and physiological markers to provide a more comprehensive understanding of performance differentiation [2].

This study demonstrates strengths in its comprehensive evaluation of anthropometric, training, performance, and technical variables, offering a holistic view of the factors distinguishing adolescent basketball players of different competitive levels. The use of standardized and validated testing protocols enhances the reliability and comparability of findings. Additionally, the inclusion of athletes from varying levels increases the generalizability of the results. The exploration of correlations between physical attributes and basketball-specific technical skills provides valuable insights, aligning well with existing theoretical frameworks.

The findings also offer practical applications for coaches, trainers, and talent scouts. The significant correlations between anthropometric traits, training exposure, and performance metrics with basketball technical skills underscore the need for tailored training programs that emphasize speed, agility, aerobic conditioning, and flexibility. These benchmarks can aid in identifying and nurturing talent, optimizing individual and team performance. The results also inform team selection, tactical planning, and specialized training regimens, contributing to enhanced competitive outcomes.

## 5. Conclusions

This study highlights significant differences in anthropometric, physical performance, physiological, and technical parameters between U-18 basketball players of high and low competitive levels. Higher-level players consistently exhibited superior physical attributes—including greater height, arm-span, and lower body fat percentage—alongside enhanced performance metrics such as faster sprinting, higher vertical jump ability, greater hamstring and lower back flexibility, and better intermittent endurance. They also demonstrated higher proficiency in technical skills such as dribbling and defensive sliding, reflecting the cumulative influence of training volume, competitive experience, and physical development. Rather than implying prediction, the present findings indicate that these anthropometric, performance, and technical characteristics are useful discriminators between competitive levels in youth basketball. Collectively, the results contribute to a clearer understanding of the multifactorial demands of the sport and may help inform evidence-based approaches to player development and selection in adolescent populations.

## Figures and Tables

**Table 1:** Anthropometrical and basketball activity profile data for all participants overall, HL, and LL groups, presented as M ± SD [95% CI].

Variable	HL (n = 38)	LL (n = 35)	Participants Overall (N = 73)
* Height (cm)	181.71 ± 5.43 [179.98–183.44]	178.51 ± 3.90 [177.22–179.80]	180.18 ± 4.99 [179.04–181.32]
Body mass (kg)	79.42 ± 9.27 [76.47–82.37]	76.59 ± 11.46 [72.79–80.39]	78.06 ± 10.40 [75.67–80.45]
* Body Fat (%)	17.13 ± 5.01 [15.54–18.72]	20.43 ± 6.30 [18.34–22.52]	18.71 ± 5.86 [17.37–20.05]
* Age (yr)	15.99 ± 0.37 [15.87–16.11]	15.73 ± 0.43 [15.59–15.87]	15.87 ± 0.42 [15.77–15.97]
* Arm-span (cm)	190.72 ± 7.18 [188.44–193.00]	181.15 ± 8.12 [178.46–183.84]	186.13 ± 8.99 [184.07–188.19]
* Maturity offset (yr)	1.93 ± 0.65 [1.72–2.14]	1.07 ± 0.73 [0.83–1.31]	1.52 ± 0.81 [1.33–1.71]
* Basketball experience (yr)	8.76 ± 2.45 [7.98–9.54]	5.07 ± 2.75 [4.16–5.98]	6.99 ± 3.18 [6.26–7.72]
Basketball-related training (hr·wk^−1^)	7.54 ± 1.73 [6.99–8.09]	6.73 ± 1.97 [6.08–7.38]	7.15 ± 1.88 [6.72–7.58]
Non-basketball training activities (self-reported; hr·wk^−1^) ^†^	2.13 ± 1.26 [1.73–2.53]	1.40 ± 2.17 [0.68–2.12]	1.78 ± 1.78 [1.37–2.19]
* Basketball-related training plus non-basketball training activities (hr·wk^−1^)	9.67 ± 2.19 [8.97–10.37]	8.13 ± 3.45 [6.99–9.27]	8.93 ± 2.95 [8.25–9.61]

* Significant difference between HL and LL groups at *p* ≤ 0.05. ^†^ Non-basketball physical training included structured exercise sessions such as running, cycling, resistance training, and other conditioning activities undertaken independently of basketball practice. Abbreviations—CI: confidence interval; HL: higher-level; LL: lower-level; M: mean; n: sample size; N: total sample size; SD: standard deviation.

**Table 2:** Performance and basketball technical skill profile data for all participants overall, HL, and LL groups, presented as M ± SD [95% CI].

Variable	HL (n = 38)	LL (n = 35)	Participants Overall (N = 73)
Dominant hand-grip strength (kg)	40.62 ± 6.26 [38.63–42.61]	40.93 ± 5.44 [39.13–42.73]	40.77 ± 5.84 [39.43–42.11]
Non dominant hand-grip strength (kg)	38.24 ± 6.16 [36.28–40.2]	39.94 ± 5.29 [38.19–41.69]	39.05 ± 5.78 [37.72–40.38]
* Hamstring and lower back flexibility (cm)	30.65 ± 9.09 [27.76–33.54]	21.15 ± 5.38 [19.37–22.93]	26.09 ± 8.89 [24.05–28.13]
** Counter-movement jump height (cm)	41.44 ± 4.44 [40.03–42.85]	35.28 ± 5.24 [33.54–37.02]	38.48 ± 5.72 [37.17–39.79]
* 10 m speed running (s)	1.86 ± 0.12 [1.82–1.90]	1.97 ± 0.16 [1.92–2.02]	1.91 ± 0.15 [1.88–1.94]
* 20 m speed running (s)	3.20 ± 0.14 [3.16–3.24]	3.38 ± 0.24 [3.30–3.46]	3.29 ± 0.21 [3.24–3.34]
* Yo-Yo IE1 (m)	3394.74 ± 760.47 [3152.95–3636.53]	2463.71 ± 817.77 [2192.79–2734.63]	2948.36 ± 912.32 [2739.08–3157.64]
HRmax during Yo-Yo IE1 (b·min^−1^)	200.16 ± 11.12 [196.62–203.70]	197.63 ± 8.05 [194.96–200.30]	198.95 ± 9.79 [196.70–201.20]
Shooting speed and accuracy (points)	38.53 ± 10.77 [35.11–41.95]	37.37 ± 4.68 [35.82–38.92]	37.97 ± 8.38 [36.05–39.89]
Passing accuracy (points)	24.84 ± 2.38 [24.08–25.60]	25.97 ± 7.89 [23.36–28.58]	25.38 ± 5.71 [24.07–26.69]
* 10 m speed dribbling (s)	1.93 ± 0.12 [1.89–1.97]	2.06 ± 0.15 [2.01–2.11]	1.99 ± 0.15 [1.96–2.02]
* 20 m speed dribbling (s)	3.33 ± 0.18 [3.27–3.39]	3.57 ± 0.27 [3.48–3.66]	3.44 ± 0.26 [3.38–3.50]
* Maneuver dribbling (s)	16.74 ± 1.20 [16.36–17.12]	18.90 ± 1.11 [18.53–19.27]	17.78 ± 1.58 [17.42–18.14]
* Defensive sliding (s)	9.89 ± 0.62 [9.69–10.09]	10.65 ± 0.71 [10.41–10.89]	10.25 ± 0.76 [10.08–10.42]

* Significant difference between HL and LL groups at *p* ≤ 0.001. ** Significant difference between HL and LL groups at *p* ≤ 0.01. Abbreviations—CI: confidence interval; HL: higher-level; HRmax: maximal heart rate; LL: lower-level; M: mean; n: sample size; N: total sample size; SD: standard deviation; Yo-Yo IE1: Yo-Yo Intermittent Endurance Level 1 test.

## Data Availability

The data supporting the findings of this study are presented as a Supplementary Materials.

## Supplementary Materials

The following supporting information can be downloaded at: https://www.mdpi.com/article/10.3390/sports14010027/s1, Table S1: raw data.
